# Immunoprotective Effects of Two Histone H2A Variants in the Grass Carp Against *Flavobacterium columnare* Infection

**DOI:** 10.3389/fimmu.2022.939464

**Published:** 2022-07-11

**Authors:** Yuan Yuan Yang, Si Yao Zheng, Hong Fang, Xiao Man Wu, Jie Zhang, Ming Xian Chang

**Affiliations:** ^1^ State Key Laboratory of Freshwater Ecology and Biotechnology, Institute of Hydrobiology, Chinese Academy of Sciences, Wuhan, China; ^2^ College of Advanced Agricultural Sciences, University of Chinese Academy of Sciences, Beijing, China; ^3^ Innovation Academy for Seed Design, Chinese Academy of Sciences, Wuhan, China

**Keywords:** grass carp, histone H2A variants, immunoprotective effect, *Saccharomyces cerevisiae*, *Flavobacterium columnare*

## Abstract

In teleost fish, the nucleotide polymorphisms of histone H2A significantly affect the resistance or susceptibility of zebrafish to *Edwardsiella piscicida* infection. Whether histone H2A variants can enhance the resistance of grass carp to *Flavobacterium columnare* infection remains unclear. Here, the effects of 7 previously obtained variants (gcH2A-1~gcH2A-7) and 5 novel histone H2A variants (gcH2A-11, gcH2A-13~gcH2A-16) in response to *F. columnare* infection were investigated. It was found that these histone H2A variants could be divided into type I and II. Among them, 5 histone H2A variants had no any effects on the *F. columnare* infection, however 7 histone H2A variants had antibacterial activity against *F. columnare* infection. The gcH2A-4 and gcH2A-11, whose antibacterial activity was the strongest in type I and II histone H2A variants respectively, were picked out for yeast expression. Transcriptome data for the samples from the intestines of grass carp immunized with the engineered *Saccharomyces cerevisiae* expressing PYD1, gcH2A-4 or gcH2A-11 revealed that 5 and 12 immune-related signaling pathways were significantly enriched by gcH2A-4 or gcH2A-11, respectively. For the engineered *S. cerevisiae* expressing gcH2A-4, NOD-like receptor and Toll-like receptor signaling pathways were enriched for up-regulated DEGs. Besides NOD-like receptor and Toll-like receptor signaling pathways, the engineered *S. cerevisiae* expressing gcH2A-11 also activated Cytosolic DNA-sensing pathway, RIG-I-like receptor signaling pathway and C-type lectin receptor signaling pathway. Furthermore, grass carp were immunized with the engineered *S. cerevisiae* expressing PYD1, gcH2A-4 or gcH2A-11 for 1 month and challenged with *F. columnare.* These grass carp immunized with gcH2A-4 or gcH2A-11 showed lower mortality and fewer numbers of *F. columnare* than did the control group. All these results suggest that gcH2A-4 and gcH2A-11 play important roles in evoking the innate immune responses and enhancing disease resistance of grass carp against *F. columnare* infection.

## Highlights

1. The obtained histone H2A variants from grass carp could be divided into type I and II.
2. Grass carp immunized with gcH2A-4 or gcH2A-11 had higher innate immune responses than the control group.
3. Compared with gcH2A-4, gcH2A-11 can activate more PRRs-mediated signaling pathways.
4. gcH2A-4 or gcH2A-11 can effectively enhance disease resistance of grass carp against *F. columnare* infection.

## Introduction

Histone H2A is one of the important components of chromosome nucleosome. Among the four core histones, variants of histone H2A and H3 are the most common. The length and sequence of the C-terminal tail of histone H2A variant differ from that of conventional histone H2A (1). The histone H2A variant H2A.X has a high content in lower eukaryotes, which can be easily distinguished from canonical H2A by the characteristic C-terminal motif SQEF (2). The N-terminal 120 amino acids of H2A.X are almost identical with that of conventional H2A, while the C-terminal 22 amino acids of H2A.X have no homology with other known vertebrate H2A protein sequences (3). The C-terminal tail of histone H2A variant H2A.Z is relatively short, and the homology of H2A.Z between different species is higher than that of conventional histone H2A within the same organism (4). The histone H2A variant macroH2A has the longest C-terminal tail and is the only histone with three domains. Histone macroH2A, nearly three times as large as that of conventional histone H2A, contains a large non-histone region (C-terminal macro domain) and a region that resembles a full length H2A (5, 6). The histone H2A variant H2A.Bbd, whose C-terminal tail is missing, is currently found only in mammals (2, 7). These histone H2A variants play important roles in regulating chromatin structure, the control of gene transcription, cell division, ontogeny and other biological processes (8, 9).

The bactericidal activity of histone-derived antimicrobial peptide has attracted extensive attention from researchers. Parasin I, an antimicrobial peptide cleaved off from the N-terminal of catfish histone H2A, showed strong antimicrobial activity (10). However, the N-terminal amino acid sequence of Parasin I significantly affected the antibacterial properties. Deletion of the lysine residue from position 1 at the N-terminal resulted in loss of antimicrobial activity of Parasin I. Compared to parasin I, the removal of the C-terminal residues 18~19 or 16~19 slightly increased the antimicrobial activity of the truncated peptide (11). In addition, several studies have shown that the complete histone H2A has antibacterial activity (12–14). However due to its larger molecular weight than antibacterial peptide, histone H2A may have certain defects in its application as an antimicrobial agent (15).

Our previous study has shown that multiple single nucleotide polymorphisms (SNPs) exist in the open reading frame of histone H2A from zebrafish and grass carp (*Ctenopharyngodon idellus*), and the nucleotide polymorphisms of histone H2A significantly affect the resistance or susceptibility of zebrafish and grass carp to *E. piscicida* infection (13). In zebrafish, the amino acid sequences of histones H2A-1, H2A-3 and H2A-4 variants are identical. The overexpression of histone H2A-1 significantly inhibited the proliferation of *E. piscicida in vivo*, whereas promoted the proliferation of *E. piscicida* for histones H2A-3 and H2A-4. In grass carp, the amino acid sequences of gcH2A-2 and gcH2A-6 variants are identical. The overexpression of histone gcH2A-2 significantly inhibited the proliferation of *E. piscicida* in CIK cells, but no obvious effect for gcH2A-6 (13). Interesting, zebrafish histone H2A variant zfH2A-6 could interact with antibacterial pattern recognition receptor NOD1, and cooperate with NOD1 to inhibit the proliferation of *Streptococcus agalactiae* (14).

*F. columnare* is an important fish pathogen, which can infect almost all freshwater fishes and cause fish columnar disease. In China, fish columnar disease is also known as bacterial rot gill disease, and causes significant losses for many important freshwater economic fish such as mandarin fish (*Siniperca chuatsi*), yellow catfish (*Pelteobagrus fulvidraco*) and the four major Chinese carps (16, 17). The effect of piscine histone H2A variant in *F. columnare* infection remains unclear. In grass carp, we obtained 5 novel histone H2A variants, which was named as gcH2A-11, gcH2A-13~gcH2A-16. Here, we describe the functional characterization of 7 previously obtained variants (gcH2A-1~gcH2A-7) and 5 novel histone H2A variants in response to *F. columnare* infection *in vitro*. Furthermore, the present study also reveal the immunoprotective effects of the engineered *S. cerevisiae* expressing gcH2A-4 and gcH2A-11 in the grass carp.

## Materials and Methods

### Experimental Fish

Healthy grass carps (mean weight 10 ± 1 g) were obtained from Chongqing, China. Fish were acclimatized in aerated freshwater with temperature maintained at 25 ± 2°C for two weeks, and fed with a commercial pelleted diet at 3% body weight per day through-out the study. All animal experiments were conducted in accordance with the Guiding Principles for the Care and Use of Laboratory Animals and were approved by the Institute of Hydrobiology, Chinese Academy of Sciences.

### Cells, Bacterial Strain and Antibodies

CIK (*C. idellus* kidney) cells were grown in MEM supplemented with 10% FBS. The wild type *F. columnare* G4 was obtained from professor Pin Nie’s lab (Institute of hydrobiology, Chinese academy of sciences), and grown at 28°C in Shieh medium or on Shieh agar. The anti-V5 mouse monoclonal antibody was purchased from Thermo Fisher Scientific.

### Plasmids Construction and Phylogenetic Analysis

The open reading frame of histone H2A variants was amplified with primer pairs H2AF/H2AR reported previously (12), and inserted into the p3×FLAG-CMV™-14 Expression Vector (Invitrogen). The cDNA template was from gill tissue of grass carp infected with *F. columnare*. The gcH2A-4-PYD1 and gcH2A-11-PYD1 plasmids were constructed using the primer pairs (**Table 1**) and inserted into the PYD1 vector (Invitrogen). All plasmid sequences were confirmed by Sanger sequencing. The gcH2A-4-PYD1 and gcH2A-11-PYD1 were verified by restriction enzymes digestion of *Bam*H I and *Eco*R I. Phylogenetic tree was constructed using the neighbor-joining (N-J) method within the MEGA (version 4.1) package.

**Table 1:** The primers used for constructing gcH2A-4-PYD1 or gcH2A-11-PYD1 and qRT-PCR validation.

Gene name	Forward primer	Reverse primer
gcH2A	CGGGATCCATGAGCGGAAGAGGCAAAAC	CGGAATTCCTTGCCTTTGGCAGCCTTC
CCL11	TTGGCCATTGCTGTTATTGA	TCAGCGTGACAACAACTTCC
TLR25	ACTCCACCATCGTTTTCCAG	ACCAAACACCATCACAAGCA
IL-8	ATGAGTCTTAGAGGTCTGGGT	ACAGTGAGGGCTAGGAGGGT
CCL35	CTCGCTTCGTCATCTTCTCC	GCTGGCTTGTCATGCTGTAA
TLR5B	TCATTGGCACACTTGTGGAT	AGTCTCGCTCCTCAAAGCAG
CD40	AGGACAGAGACTCGCCAAAA	ACAGGTCCGATCTGAGGTTG
CCL19	TTCCTGATGTTAACCGTTATTGG	CCTCTAGGCAGGATCTCCAC
TRAF3	GAGTTGTCCAGTCACCAGCA	TTCCAGCTGCCTGTTCTTTT
AP-1L	ATGAGTCTTAGAGGTCTGGGT	ATGAGTCTTAGAGGTCTGGGT
TNFα	CGCTGCTGTCTGCTTCA	CCTGGTCCTGGTTCACTC
IL-1β	AGAGTTTGGTGAAGAAGAGG	TTATTGTGGTTACGCTGGA
β-actin	GGCTGTGCTGTCCCTGTA	GGGCATAACCCTCGTAGAT

### Antibacterial Activity for H2A Variants *In Vitro*

For *in vitro* bacterial infection assays, CIK cells seeded overnight in 24-well plates at 3×10^5^ cells per well were transfected with 500 ng p3×FLAG or histone H2A variants including gcH2A-1-FLAG, gcH2A-2-FLAG, gcH2A-3-FLAG, gcH2A-4-FLAG, gcH2A-5-FLAG, gcH2A-6-FLAG, gcH2A-7-FLAG, gcH2A-11-FLAG, gcH2A-13-FLAG, gcH2A-14-FLAG, gcH2A-15-FLAG or gcH2A-16-FLAG using Lipofectamine™ 2000 (Invitrogen). After 24 h post-transfection, CIK cells were infected with *F. columnare* at a multiplicity of infection (MOI) of 50 in serum-free MEM medium for 1.5 h. At 3 and 6 hours post infection (hpi), the supernatants and cells were collected together. Then the mixture was diluted with Shieh medium and plated onto Shieh agar to calculate bacterial CFU (colony-forming units) by standard plate count method.

### Expression of Recombinant gcH2A-4 and gcH2A-11 in the *S. cerevisiae* Strain EBY100

The recombinant gcH2A-4-PYD1 and gcH2A-11-PYD1 plasmids were transformed to *S. cerevisiae* strain EBY100. The positive colonies of gcH2A-4-PYD1, gcH2A-11-PYD1 and PYD1 were grown in 2% glucose YNB−CAA liquid medium at 30°C for 48~72 h. When the OD_600_ value of the culture reached between 2.0~5.0, yeast were centrifuged at 4,000 rpm/min at 4°C, and resuspended in YNB-CAA medium containing 2% galactose with the OD_600_ = 1. Then, the culture of gcH2A-4-PYD1, gcH2A-11-PYD1 and PYD1 were induced in YNB-CAA medium containing 2% galactose at 20°C for 24 h. Total protein was extracted and analyzed by 10% SDS-PAGE. The PVDF membrane was washed and incubated with anti-V5 mouse monoclonal antibody (1: 5000) overnight at 4°C. After washing with TBST, the membrane was next incubated with Goat-anti-mouse Ig-HRP conjugate secondary Ab (1: 5000) for 1 h at room temperature. The bands were detected using Pierce ECL Western Blotting Substrate and ECL Western blot system (LAS-4000mini).

### Immunization of Grass Carp and Illumina Deep Sequencing

The yeast pellets of gcH2A-4-PYD1, gcH2A-11-PYD1 and PYD1 was resuspended in 10 mL PBS buffer with the cell density of 1.2×10^8^ cells/mL. Gass carp were intraperitoneally injected with 200 μL of the engineered *S. cerevisiae* expressing gcH2A-4, gcH2A-11 or PYD1. After the immunization for 7 days, the intestines from 3 fish each group were flash-frozen in liquid nitrogen and stored at -80°C for transcriptome sequencing and qRT-PCR verification.

According to the methods from our previous report (18), cDNA library construction and illumina deep sequencing were performed. Briefly, total RNA was extracted using the TRIzol^®^ Reagent, and the mRNA was isolated using oligo (dT) magnetic beads with the TruseqTM RNA sample prep Kit (Illumina, California, USA). Raw reads were produced by an Illumina Hiseq4000 instrument, and the raw sequences were deposited at NCBI Gene Expression Omnibus (GEO) database under the accession number GSE201422. Differential expression gene (DEG) analysis of two groups was performed using the DESeq R package. Genes with a fold change ≥ 1.5 and FDR ≤ 0.05 (adjusted *P* value ≤ 0.05) were defined as significant DEGs. KEGG pathway enrichment analysis was conducted with KEGG Orthology-Based Annotation System, with the Bonferroni correction used to adjust *p*-values. KEGG pathways with the corrected *p*-value (Q-Value) < 0.05 were considered significantly enriched.

### Experimental Validation by qRT-PCR

The same RNA samples from the intestines of the engineered *S. cerevisiae* expressing gcH2A-4, gcH2A-11 or PYD1 were used for qRT-PCR validation of the RNA-seq analysis. RNase-free DNase I (Thermo) was used to remove genomic DNA remnants at 37°C for 30 min. The cDNA was synthesized using the RevertAid™ First Strand cDNA Synthesis Kit (Thermo Fisher Scientific). qRT-PCR analysis was performed using Fast SYBR Green PCR Master mix (Bio-Rad) to validate the DEGs involved in the Toll like receptor pathway under the following program: 3 min at 95°C , followed by 45 cycles of 15 s at 94°C, 15 s at 58°C and 30 s at 72°C Those DEGs for validation include CCL11 (CI01000039_04065787_04066284.path1), TNFα (CI01000054_10082299_10083956.path1), AP−1L (CI01000205_00077363_00081315.path1), CD40 (CI01000001_01357469_01365104.path1), TLR5B (CI01000029_07503905_07506541.path1), IL−8 (CI01000300_03901275_03902800.path1), CCL35 (CI01000145_00951480_00956106.path1), IL−1β (CI01000204_00402898_00405195.path1), TRAF3 (CI01000189_02080315_02089268.path1), CCL19 (CI01000343_00865224_00865808.path1), TLR25 (CI01000020_05132989_05135436.path1). Grass carp β-actin was used as internal control. The relative fold changes were calculated by comparison to the corresponding controls using the comparative CT (2^−△△Ct^) method. All primers used for qRT-PCR are shown in **Table 1**.

### Immunization and Infection of Grass Carp

To analyze the protective effects of the engineered *S. cerevisiae* expressing gcH2A-4 and gcH2A-11 in the grass carp against *F. columnare* infection, grass carp were intraperitoneally injected with 200 μL of the engineered *S. cerevisiae* expressing gcH2A-4, gcH2A-11 or PYD1 (1.2×10^8^ CFU/mL) for two times with the interval for two weeks. After the immunization for 30 days, 30 fish per group were infected with 4.0 × 10^6^ CFU/mL *F. columnare* for 4 h in total volume of 18 L barrel, and next maintained in 70 L barrel with the volume of 30 L aerated sterile water.

Thirty fish each group were used for survival assays. The number of surviving grass carp was counted daily for 7 days post-infection (dpi). GraphPad Prism 7 was used to generate survival curves.

The gill tissues from 3 fish each group were collected at 1 and 2 dpi, and used for measuring bacterial burden. The gills were rinsed and lysed in 1 ml PBS using a glass homogenizer. Serial dilutions of the homogenates were plated onto Shieh agar, and CFU were counted after 24 h of incubation at 28°C.

### Statistical Analysis

Statistical analysis and graphs were performed and produced using Graphpad Prism 7.0 software. Data from qRT-PCR and antibacterial activities for H2A variants *in vitro* and *in vivo* are presented as mean and SEM. The significance of results was analyzed by a two-tailed Student’s t-test (*p < 0.05; **p < 0.01). The log-rank test was used to test differences in survival between the grass carp immunized with PYD1 and grass carp immunized with gcH2A-4-pYD1 or gcH2A-11-pYD1.

## Results

### Features of Grass Carp Histone H2A Variants

**Figure 1:**
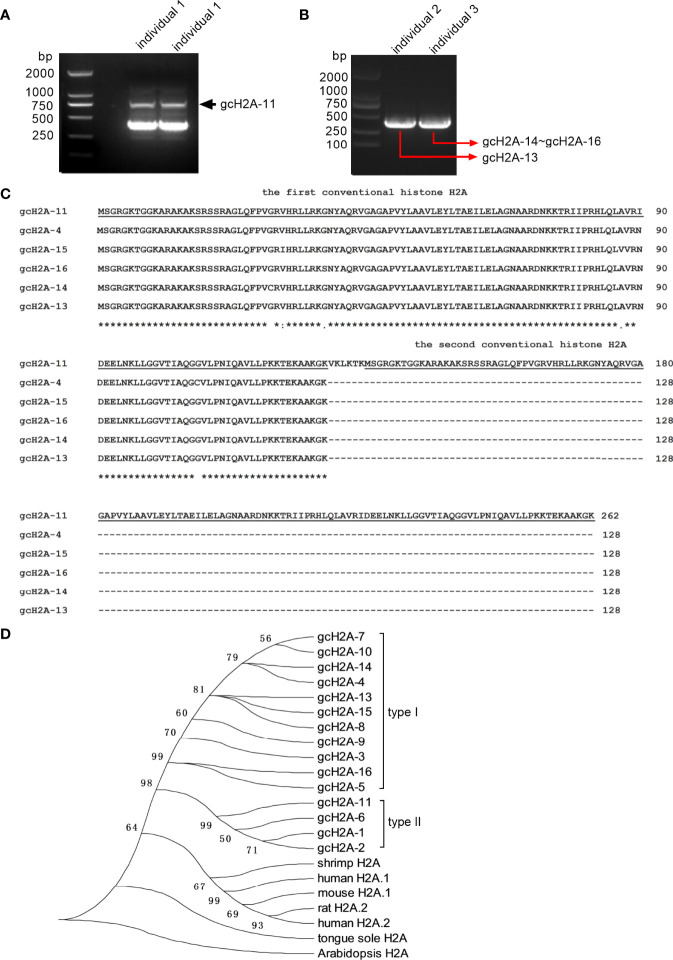
The cloning and sequence analysis of grass carp histone H2A variants. **(A)** The cloning of gcH2A-11 from the gills of grass carp individual 1 infected with *F. columnare*. **(B)** The cloning of gcH2A-13 and gcH2A-14~16 from the gills of grass carp individual 2 and 3 infected with *F. columnare*, respectively. **(C)** Sequence alignments of gcH2A-4, gcH2A-11 and gcH2A-13~16. The conventional histone H2A region is underlined. **(D)** Phylogenetic analysis of grass carp histone H2A variants, and histone H2A from other species including shrimp H2A (GenBank accession number: XM_037921631), human H2A.1 (GenBank accession number: M60752), mouse H2A.1 (GenBank accession number: M33988), rat H2A.2 (GenBank accession number: JX661509), human H2A.2 (GenBank accession number: L19779), tongue sole H2A (GenBank accession number: KU904500) and Arabidopsis H2A (GenBank accession number: AK228272).

The grass carp histone H2A variants gcH2A-1~gcH2A-10 have been reported in our previous study, which were obtained from the mixed cDNA templates (13). Here, we found 5 new histone H2A variant (named as gcH2A-11, gcH2A-13~gcH2A-16), which were obtained from 3 grass carp infected with *F. columnare*. gcH2A-11 variant was obtained from the gill tissues of grass carp individual 1, gcH2A-13 variant from the gill tissues of grass carp individual 2, gcH2A-14~ gcH2A-16 variants from the gill tissues of grass carp individual 3 (**Figures 1A, B**).

The amino acid sequences of gcH2A-11 (GenBank accession number: ON184271) and gcH2A-13 ~ gcH2A-16 (GenBank accession numbers: ON323663~ON323666) were analyzed. It was found that gcH2A-11 encoded two identical conventional H2A sequences, which were linked by six amino acids. Comparison among the first conventional H2A sequences of gcH2A-11 and the complete H2A sequences of gcH2A-4, gcH2A-13 ~ gcH2A-16 revealed only one amino acid site difference between any two variants (**Figure 1C**).

Based on nucleotide sequences of the first conventional H2A sequences of gcH2A-11 and the complete H2A sequences of gcH2A-1 ~ gcH2A-10, gcH2A-13 ~ gcH2A-16, phylogenetic trees were constructed using neighbor-joining method. The grass carp histone H2A variants are obviously divided into two classes. Type I contains more H2A variants including gcH2A-3~gcH2A-5, gcH2A-7~gcH2A-10 and gcH2A-13~gcH2A-16. Type II contains gcH2A-1, gcH2A-2, gcH2A-6 and gcH2A-11 (**Figure 1D**).

### Antibacterial Activities of Grass Carp Histone H2A Variants

**Figure 2:**
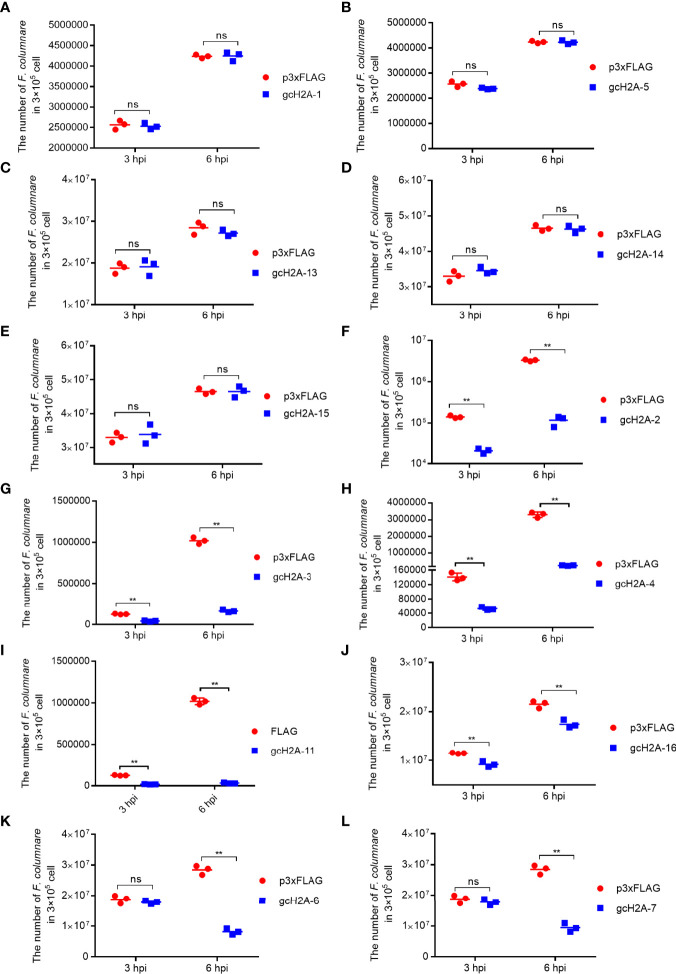
The effects of grass carp histone H2A variants in bacterial infection. **(A–E)** No significant effects of gcH2A-1, gcH2A-5, gcH2A-13, gcH2A-14 and gcH2A-15 on the proliferation of *F. columnare in vitro*. **(F–J)** The antibacterial effects of gcH2A-2, gcH2A-3, gcH2A-4, gcH2A-11 and gcH2A-16 both at 3 hpi and 6 hpi. **(K, L)** The antibacterial effects of gcH2A-6 and gcH2A-7 at 6 hpi. Data represented means ± SEM (n=3), and were tested for statistical significance. **p < 0.01; ns, not significant. The asterisk above the bracket indicates statistical significance between the two groups connected by the bracket.

To evaluate the roles of grass carp histone H2A variants in the bacterial infection of *F. columnare*, CIK cells transfected with the empty plasmid FLAG or gcH2A variant-FLAG were infected with *F. columnare*. Compared with the control group transfected with the p3×FLAG empty plasmid, gcH2A-1, gcH2A-5, gcH2A-13, gcH2A-14 and gcH2A-15 had no significant effect on the proliferation of *F. columnare* (**Figures 2A–E**), but significantly inhibited the proliferation of *F. columnare* at 3 and 6 hpi for gcH2A-2, gcH2A-3, gcH2A-4, gcH2A-11 and gcH2A-16 (**Figures 2F–J**). At 3 hpi, the numbers of *F. columnare* in the control group transfected with empty plasmid were 6.6-fold, 3.0-fold, 2.7-fold, 6.9-fold and 1.3-fold than that transfected with gcH2A-2, gcH2A-3, gcH2A-4, gcH2A-11 and gcH2A-16 respectively. At 6 hpi, the numbers of *F. columnare* in the control group transfected with empty plasmid were 28.3-fold, 6.2-fold, 12.9-fold, 30.6-fold and 1.2-fold than that transfected with gcH2A-2, gcH2A-3, gcH2A-4, gcH2A-11 and gcH2A-16 respectively. gcH2A-6 and gcH2A-7 significantly inhibited the proliferation of *F. columnare* only at 6 hpi (**Figures 2K, L**). The numbers of *F. columnare* in the control group transfected with empty plasmid were 3.5-fold than that transfected with gcH2A-6, and 3.0-fold than that transfected with gcH2A-7. Together, these data suggest that gcH2A-11, whose size of amino acid sequences is twice that of other histones H2A variants, has the highest antibacterial activity *in vitro*.

### Construction and Expression of Recombinant *S. cerevisiae*

**Figure 3:**
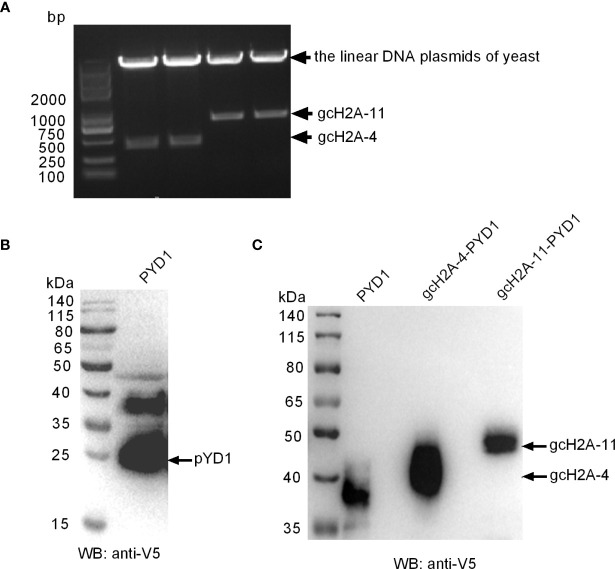
The recombinant expressions of gcH2A-4 and gcH2A-11 in the *S. cerevisiae* strain. **(A)** Validation of the recombinant gcH2A-4−PYD1 and gcH2A-11−PYD1 plasmids using *Bam*H I/*Eco*R I double restriction enzyme digestion. **(B)** Western Blotting of the cell lysates transformed with the PYD1 empty plasmid. **(C)** Western Blotting of the cell lysates transformed with PYD1, gcH2A-4−PYD1 or gcH2A-11−PYD1.

Among the obtained 7 gcH2A variants with antibacterial roles against *F. columnare* infection, the effects of gcH2A-4 and gcH2A-11 in inhibiting the proliferation of *F. columnare* is the strongest in type I and type II histone H2A variants, respectively. Therefore, gcH2A-4 and gcH2A-11 were selected for yeast expression. The recombinant plasmids (gcH2A-4−PYD1 and gcH2A-11−PYD1) were constructed successfully and validated by *Bam*H I/*Eco*R I double restriction enzyme digestion (**Figure 3A**). The recombinant gcH2A-4−PYD1 and gcH2A-11−PYD1 were integrated into downstream of the GAL1 and T7 promoters of *S. cerevisiae* strain, and the expressions of gcH2A-4−PYD1 and gcH2A-11−PYD1 were induced by D-galactose. Analysis of cell lysates by 10% SDS-PAGE electrophoresis showed that an obvious heteroband between 35~40 kDa was sometimes detected when the cell lysates transformed with the PYD1 empty plasmid were detected with anti-V5 antibody, in addition to the predicted 25 kDa band of PYD1 protein (**Figure 3B**). Only a single band with an approximate 40 kDa was detected in the cell lysates transformed with gcH2A-4−PYD1 plasmid, and an approximate 50 kDa observed in the cell lysates transformed with gcH2A-11−PYD1 plasmid (**Figure 3C**). The protein bands corresponding to gcH2A-4−PYD1 and gcH2A-11−PYD1 did not exist in the cell lysates transformed with the PYD1 empty plasmid. These data from Western Blotting suggest that gcH2A-4−PYD1 and gcH2A-11−PYD1 have been expressed successfully in *S. cerevisiae* strain.

### Analysis of Differentially Expressed Genes Regulated by the Engineered *S. cerevisiae* Expressing gcH2A-4 and gcH2A-11

**Figure 4:**
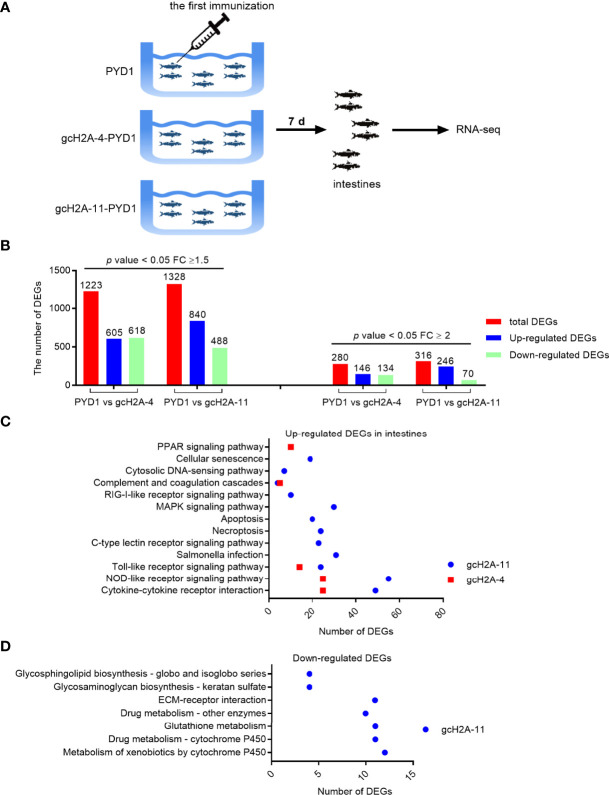
Differentially expressed genes and significantly enriched KEGG pathways in grass carp regulated by the engineered *S. cerevisiae* expressing gcH2A-4 and gcH2A-11. **(A)** The schematic diagram for the immunized grass carp used for illumina deep sequencing. **(B)** The number of DEGs regulated by the engineered *S. cerevisiae* expressing gcH2A-4 and gcH2A-11. **(C)** The significantly enriched KEGG pathways for up-regulated DEGs in intestines regulated by the engineered *S. cerevisiae* expressing gcH2A-4 and gcH2A-11. **(D)** The significantly enriched KEGG pathways for down-regulated DEGs in intestines regulated by the engineered *S. cerevisiae* expressing gcH2A-4 and gcH2A-11.

To reveal the possible immunoregulatory functions of the engineered *S. cerevisiae* expressing gcH2A-4 and gcH2A-11 on the grass carp, the intestines from the immunized grass carp were collected at the first immunization for 7 days and used for transcriptome sequencing (**Figure 4A**). Based on the *p* value < 0.05 and Fold Change (FC) ≥ 1.5, in all 1223 DEGs (605 up- and 618 down-regulated) were found in PYD1 vs gcH2A-4 group, 1328 DEGs (840 up- and 488 down-regulated) found in PYD1 vs gcH2A-11 group. Based on the *p* value < 0.05 and Fold Change (FC) ≥ 2, in all 280 DEGs (146 up- and 134 down-regulated) were found in PYD1 vs gcH2A-4 group, 316 DEGs (246 up- and 70 down-regulated) found in PYD1 vs gcH2A-11 group (**Figure 4B**).

KEGG enrichment analysis was used to determine the significantly enriched pathways regulated by the engineered *S. cerevisiae* expressing gcH2A-4 and gcH2A-11. For the engineered *S. cerevisiae* expressing gcH2A-4, five pathways including PPAR signaling pathway, Complement and coagulation cascades, Toll-like receptor signaling pathway, NOD-like receptor signaling pathway and Cytokine-cytokine receptor interaction were significantly enriched for the up-regulated DEGs based on that corrected q value < 0.05. For the engineered *S. cerevisiae* expressing gcH2A-11, twelve pathways including Cytokine-cytokine receptor interaction, NOD-like receptor signaling pathway, Toll-like receptor signaling pathway, Salmonella infection, C-type lectin receptor signaling pathway, Necroptosis, Apoptosis, MAPK signaling pathway, RIG-I-like receptor signaling pathway, Complement and coagulation cascades and Cytosolic DNA-sensing pathway were significantly enriched for the up-regulated DEGs based on that corrected q value < 0.05 (**Figure 4C**). For the down-regulated DEGs, no any pathway or no immune-related pathways were significantly enriched for the engineered *S. cerevisiae* expressing gcH2A-4 or gcH2A-11, respectively (**Figure 4D**). Collectively, these results suggest that grass carp immunized with gcH2A-4 or gcH2A-11 had higher innate immune response than the control group.

### The Expression Patterns of DEGs Involved in PRRs-Mediated Signaling Pathways

**Figure 5:**
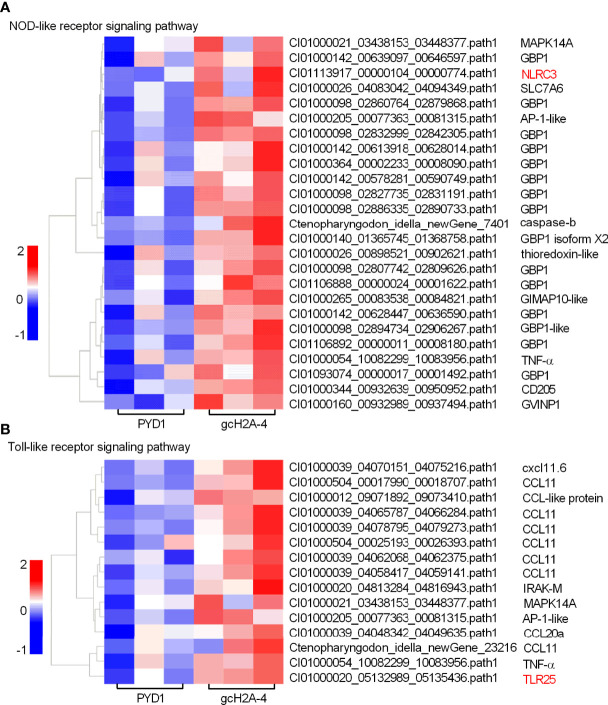
The effects of the engineered *S. cerevisiae* expressing gcH2A-4 on the expression patterns of DEGs involved in PRRs-mediated signaling pathways. **(A)** The gene cluster for DEGs involved in the NOD-like receptor signaling pathway for the samples from the intestines of grass carp after 7 days of the first immunization. **(B)** The gene cluster for DEGs involved in the Toll-like receptor signaling pathway for the samples from the intestines of grass carp after 7 days of the first immunization. A color key denotes the gradient scale of gene expression from low (blue) to high (red) degrees. The receptors of NLR and TLR are underlined.

For the engineered *S. cerevisiae* expressing gcH2A-4, the expression patterns of DEGs involved in PRRs-mediated signaling pathways including NOD-like receptor and Toll-like receptor signaling pathways were examined. Among 25 DEGs involved in NOD-like receptor signaling pathway, 15 DEGs were guanylate-binding protein 1 (*GBP1*). For the PRRs involved in NOD-like receptor signaling pathway, only NLRC3 gene was induced by gcH2A-4 (**Figure 5A**). Among 15 DEGs involved in Toll-like receptor signaling pathway, 10 DEGs were chemokine or chemokine ligand. For the PRRs involved in Toll-like receptor signaling pathway, only TLR25 was induced by gcH2A-4 (**Figure 5B**).

**Figure 6:**
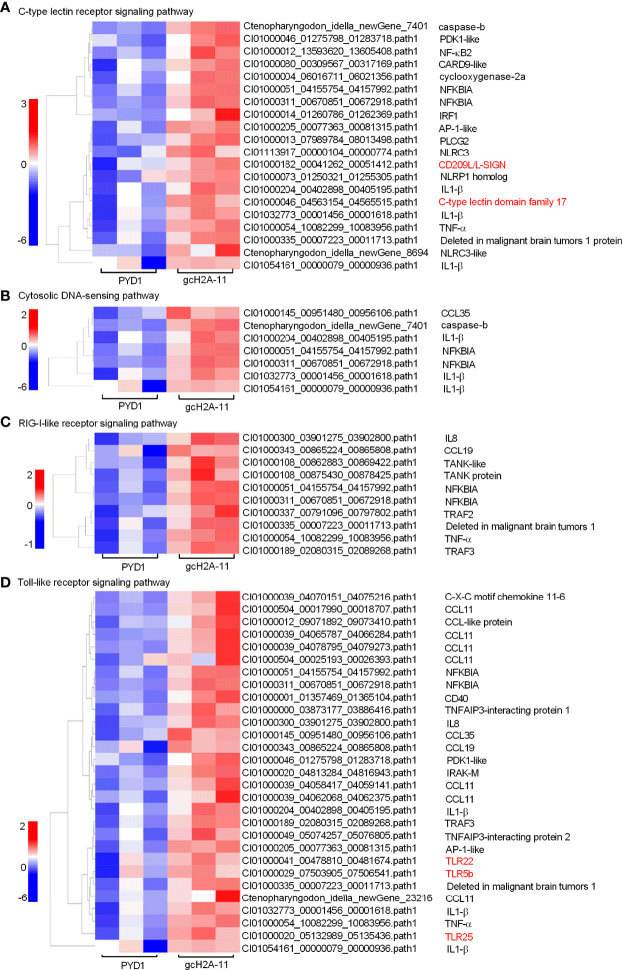
The effects of the engineered *S. cerevisiae* expressing gcH2A-11 on the expression patterns of DEGs involved in PRRs-mediated signaling pathways. **(A)** The gene cluster for DEGs involved in the C-type lectin receptor signaling pathway for the samples from the intestines of grass carp after 7 days of the first immunization. **(B)** The gene cluster for DEGs involved in the Cytosolic DNA-sensing pathway for the samples from the intestines of grass carp after 7 days of the first immunization. **(C)** The gene cluster for DEGs involved in the RIG-I-like receptor signaling pathway for the samples from the intestines of grass carp after 7 days of the first immunization. **(D)** The gene cluster for DEGs involved in the Toll-like receptor signaling pathway for the samples from the intestines of grass carp after 7 days of the first immunization. A color key denotes the gradient scale of gene expression from low (blue) to high (red) degrees. The receptors of CLR and TLR are underlined.

**Figure 7:**
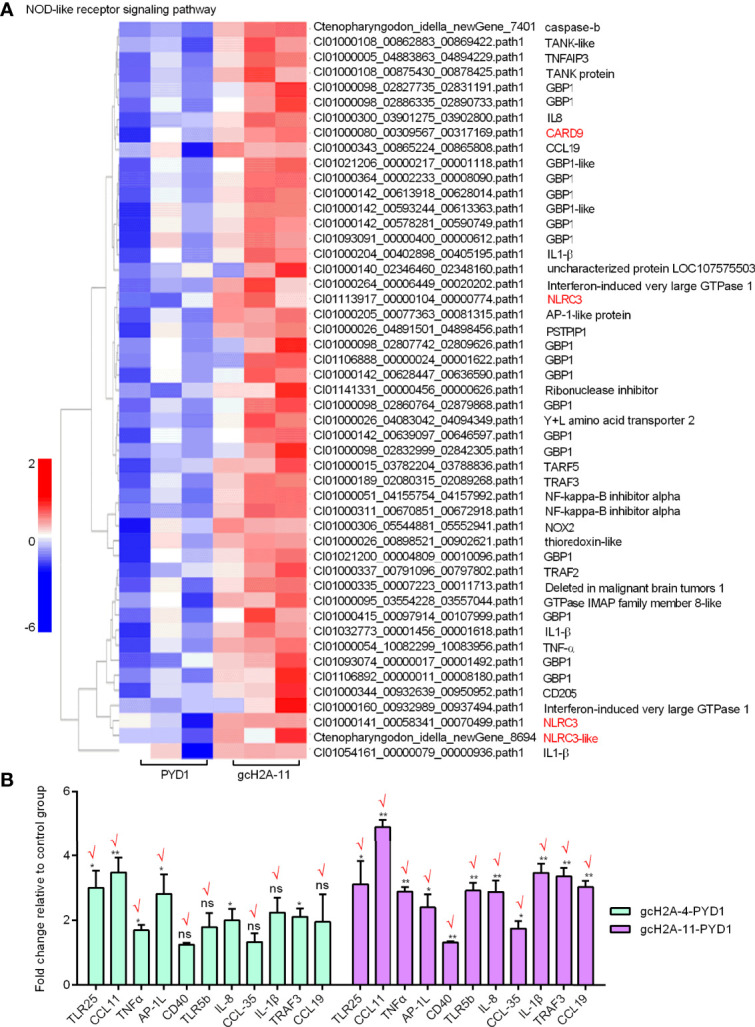
Validation of transcriptome data by qRT-PCR. **(A)** The gene cluster for DEGs involved in the NOD-like receptor signaling pathway for the samples from the intestines of grass carp after 7 days of the first immunization using the engineered *S. cerevisiae* expressing gcH2A-11. **(B)** Validation of transcriptome data by qRT-PCR for 11 DEGs involved in the Toll like receptor pathway. Data represented means ± SEM (n=3), and were tested for statistical significance. *p < 0.05; **p < 0.01; ns, not significant.

For the engineered *S. cerevisiae* expressing gcH2A-11, the expression patterns of DEGs involved in five significantly enriched PRRs-mediated signaling pathways were examined. For C-type lectin receptor signaling pathway (20 DEGs), CD209L and C-type lectin domain family 17 were induced by gcH2A-4 (**Figure 6A**). For Cytosolic DNA-sensing pathway (7 DEGs) and RIG-I-like receptor signaling pathway (10 DEGs), no any PRR was regulated by gcH2A-11 (**Figures 6B, C**). For Toll-like receptor signaling pathway (29 DEGs), 3 PRRs including TLR5b, TLR22 and TLR25 were induced by gcH2A-11 (**Figure 6D**). For NOD-like receptor signaling pathway (49 DEGs), 4 DEGs including CARD9, NLRC3 and NLRC3-like genes were induced by gcH2A-11 (**Figure 7A**). Except for CCL35, these DEGs involved in Cytosolic DNA-sensing pathway and RIG-I-like receptor signaling pathway were also involved in NOD-like receptor signaling pathway.

The expression of 11 candidate DEGs was confirmed by qRT-PCR in the engineered *S. cerevisiae* expressing PYD1 empty plasmid, gcH2A-4-PYD1 or gcH2A-11-PYD1. Except for TRAF3, the expression of other 10 DEGs for PYD1 vs gcH2A-4 group agreed with their changes determined by RNA-seq. Furthermore, the expression of all 11 examined DEGs for PYD1 vs gcH2A-11 group agreed with their significant changes determined by RNA-seq (**Figure 7B**). In all, 21/22 (95.5%) consistency existed between qRT-PCR and RNAseq.

### Protective Effects of the Engineered *S. cerevisiae* Expressing gcH2A-4 and gcH2A-11 in the Grass Carp Against *F. columnare* Infection

**Figure 8:**
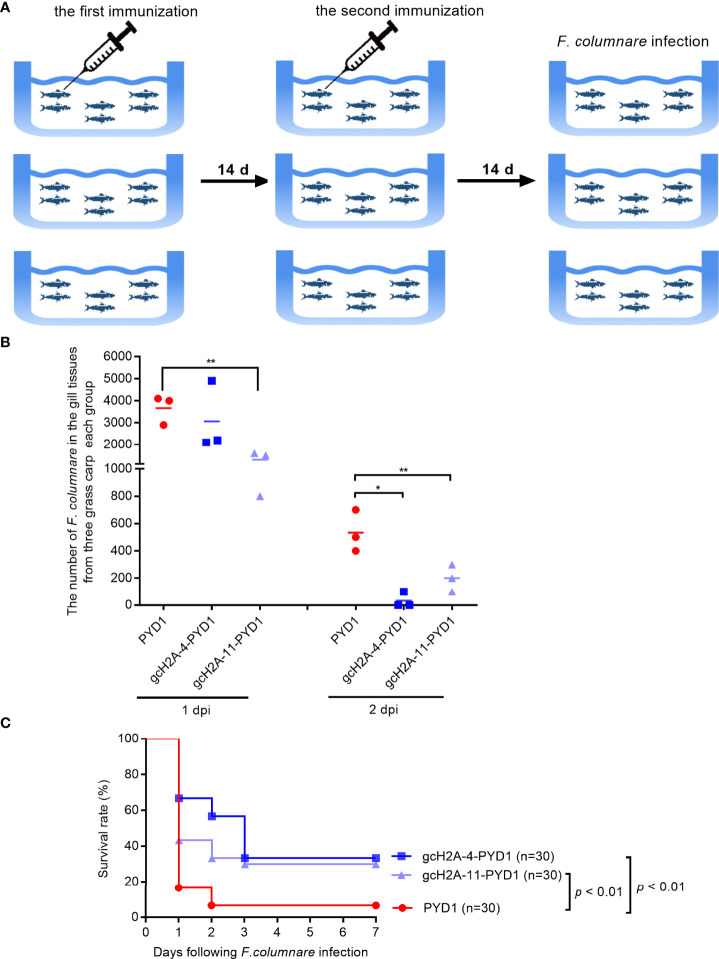
The effects of the engineered *S. cerevisiae* expressing gcH2A-4 and gcH2A-11 on the disease resistance of grass carp against *F. columnare* infection. **(A)** The schematic diagram for the immunized grass carp used for survival analysis. **(B)** The effects of the engineered *S. cerevisiae* expressing gcH2A-4 and gcH2A-11 on the bacteria proliferation of *F. columnare* Data represented means ± SEM (n=3), and were tested for statistical significance. *p < 0.05; **p < 0.01. **(C)** The effects of the engineered *S. cerevisiae* expressing gcH2A-4 and gcH2A-11 on the survival of grass carp in response to *F. columnare* infection.

Grass carp in all the immunized groups with the engineered *S. cerevisiae* expressing gcH2A-4 and gcH2A-11 for 30 days were challenged with *F. columnare* by immersion (**Figure 8A**). At 1 and 2 dpi, gill tissues of grass carp were taken for bacteria counting. The results from bacterial colony counting showed that the immunized groups with the engineered *S. cerevisiae* expressing gcH2A-4 and gcH2A-11 have the lower bacterial loads compared with the PYD1 control group, especially at 2 dpi (**Figure 8B**). The dead fish was examined every day. The death of grass carp caused by *F. columnare* infection mainly occurred on the first day. The average mortality of the PYD1 control group was 83.33% at 1 dpi, 33.33% for the immunized group with the engineered *S. cerevisiae* expressing gcH2A-4, 56.67% for the immunized group with the engineered *S. cerevisiae* expressing gcH2A-11. At 2 dpi, the PYD1 control group had a mean cumulative mortality of 93.33%, with no further deaths thereafter. The mean cumulative mortality was 43.33% at 2 dpi, 66.67% at 3 dpi in the immunized group with the engineered *S. cerevisiae* expressing gcH2A-4, and there was no further death thereafter. For the immunized group with the engineered *S. cerevisiae* expressing gcH2A-11, the mean cumulative mortality was 66.67% at 2 dpi, 70.00% at 3 dpi, and there was no further death thereafter. Overall, the protective rates of the engineered *S. cerevisiae* expressing gcH2A-4 and gcH2A-11 in the grass carp against *F. columnare* infection were 28.5% and 25%, respectively (**Figure 8C**).

## Discussion

Histones play an important role in regulating gene expression and chromatin structure. Due to differences in amino acid composition and molecular weight, histones can be divided into five types, including H2A, H2B, H3, H4 and linker histones (19). The anti-microbial properties of antimicrobial peptides derived from histone H2A have been well established, such as antimicrobial peptide teleostin obtained from marine teleost fishes *Cynoglossus semifasciatus* and *Tachysurus jella* (20), antimicrobial peptide hipposin obtained from the skin mucus of Atlantic halibut *Hippoglossus hippoglossus L (* 21 *).*, and antimicrobial peptide Parasin I obtained from the catfish *Parasilurus asotus* (10). Furthermore, several studies have shown that histone H2A itself has antibacterial activity against both Gram-positive and Gram-negative bacteria (22). Our previous studies revealed that the nucleotide polymorphisms of histone H2A significantly affect the resistance or susceptibility of zebrafish and grass carp to *E. piscicida* infection (13). In the present study, we describe the functional characterization of grass carp histone H2A variants in *F. columnare* infection. In grass carp, the amino acid sequences of gcH2A-2 and gcH2A-6 are identical. In response to *E. piscicida* infection, gcH2A-2 has an antibacterial effect, while gcH2A-6 has no effect (13). Different from *E. piscicida* infection, both gcH2A-2 and gcH2A-6 significantly inhibited the proliferation of *F. columnare*. In addition, gcH2A-7 had no effect on *E. piscicida* infection (13), but inhibited the proliferation of *F. columnare*. The effect of gcH2A-1, gcH2A-3 and gcH2A-4 in the *F. columnare* infection is similar to that in the *E. piscicida* infection. The present data together with our previous studies suggest that differences in amino acids rather than nucleotides of grass carp histone H2A variants affect the antibacterial activity of grass carp histone H2A variants against *F. columnare* infection, which is different from *E. piscicida* infection.

*S. cerevisiae* rich in beta-glucans and mannan-oligosaccharides is a well-known probiotic, which can activate the innate and adaptive immune responses and enhance the host’s resistance to pathogen infection (23, 24). *S. cerevisiae* itself is a safe yeast used as the production of ingredients for human nutrition and health, and can efficiently display biologically active proteins and exogenous DNA as vaccines (24–26). Therefore, *S. cerevisiae* was selected as the expression vector to express grass carp gcH2A−4 and gcH2A−11. After immunizations for 7 days, the results from transcriptome sequencing showed that the engineered *S. cerevisiae* expressing gcH2A-4 and gcH2A-11 can elicit the innate immune responses, especially for the PRRs-mediated signaling pathways. The correlation between histone H2A and NOD-like receptor signaling pathway has been revealed in zebrafish. The deficiency of NLR receptor NOD1 or adapter protein RIP2 involved in NOD-like receptor signaling pathway was found to impair the transcription of histone H2A (12, 14). NOD1 could colocalize with histone H2A both in the cytoplasm and cell nucleus in the case of *S. agalactiae* infection, and interact with histone H2A variant (14). Here, the engineered *S. cerevisiae* expressing gcH2A-4 and gcH2A-11 can activate NOD-like receptor signaling pathway. Interesting, NLRC3 or NLRC3-like genes rather than NOD1 or NOD2 were induced by the engineered *S. cerevisiae* expressing gcH2A-4 and gcH2A-11. Among those DEGs increased by gcH2A-4 or gcH2A-11, above than 15 DEGs are orthologs of guanylate-binding protein 1 (GBP1). In mammals, several lines of evidence show that GBP1 is interferon-stimulated gene (ISG), and is essential for autonomous host defense against intracellular pathogens (27). GBP1 can solicit host defense proteins including the phagocyte oxidase, antimicrobial peptides and autophagy effectors to kill intracellular bacteria (28), and function as a bona fide PRR for bacterial LPS through disrupting the integrity of bacterial outer membranes (29). Furthermore, GBP1 is also a major restriction factor for many viruses such as the Hepatitis E virus and Herpes Simplex virus type 1 (30, 31). The present study reveals a link between histone H2A and GBP1. The induced transcriptions of multiple GBP1 orthologs by the engineered *S. cerevisiae* expressing gcH2A-4 and gcH2A-11 suggest the possible protective effect of gcH2A-4 and gcH2A-11 in the grass carp against bacterial or viral infections.

Besides NOD-like receptor signaling pathway, Toll-like receptor signaling pathway is the common PRRs-mediated signaling pathway activated by both the engineered *S. cerevisiae* expressing gcH2A-4 and gcH2A-11. To date, 18 different TLRs have been identified in fish species. In teleost fish, TLR25 is an identified fish-specific member of the TLR1 subfamily, and is involved in activating NF-κB and type I IFNs signaling pathways and inducing the expression of proinflammatory cytokines such as TNF-α, IL1β and IL8 (32, 33). Previous studies have suggested that TLR2 and TLR4 are receptors for histones, and required for responses to all individual histones (34, 35). However in the grass carp, TLR2 and TLR4 failed to be induced by gcH2A-4 and gcH2A-11, but TLR25 increased by both the engineered *S. cerevisiae* expressing gcH2A-4 and gcH2A-11. It is interesting to further investigate whether the fish-specific TLR25 is the receptor of grass carp histone H2A. TLR22 has been found in several fish species but as a non-functional pseudogene in mammals (36). Piscine TLR22 plays critical role in restricting intracellular survival of *Aeromonas hydrophila* through the activation of TNF-α/caspase-1/IL-1β inflammatory axis (37). TLR22 is also associated with innate immunity against viral and ectoparasite infection (38, 39). In addition, more than three paralogous TLR22 genes are possessed by some teleost fishes, and present the functional diversity in ligand recognition and signal activation (40, 41). In the grass carp, the immunization with the engineered *S. cerevisiae* expressing gcH2A-11 also induced TLR22 and TNF-α/caspase-b/IL-1β, which was different from the engineered *S. cerevisiae* expressing gcH2A-4. Further work is needed to assess whether the functional correlation exists between gcH2A-11 and TLR22 subtypes in response to pathogen infection.

Besides NOD-like receptor signaling pathway and Toll-like receptor signaling pathway, the engineered *S. cerevisiae* expressing gcH2A-11 can activate C-type lectin receptor signaling pathway, Cytosolic DNA-sensing pathway and RIG-I-like receptor signaling pathway. Only most DEGs involved in Cytosolic DNA-sensing pathway and RIG-I-like receptor signaling pathway were also included in NOD-like receptor signaling pathway. The C-type lectin receptors (CLRs) are divided into 17 groups based on functional and structural characteristics, which activate NF-κB through a Syk- and CARD9-dependent pathway to induce innate immune and inflammatory responses following microbial infection (42). Among the multiple subgroups of lectin superfamily, CD209/DC-SIGN is one of these subgroups. Mammalian CD209 and CD209L serve as receptors for many viruses, bacteria and parasites such as Ebolavirus, Leishmania and *Yersinia pestis* (43–45). Here, the immune-related lectin-like receptor (C-type lectin domain family 17), CD209L, CARD9-like, NF-κB2 and NFKBIA genes are induced by the engineered *S. cerevisiae* expressing gcH2A-11 but not by the engineered *S. cerevisiae* expressing gcH2A-4, which suggest that C-type lectin domain family 17 and CD209L may recognize gcH2A-11 and activate NF-κB in the CARD9-dependent manner. A study has shown that the macrophage-expressed C-type lectin-receptor-2d (Clec2d) is able to recognize all 5 histone proteins *via* sequences in the N-terminal tail of all histones and also in the C-terminal tail of H1 (46). Since the differences in amino acids exist at the C-terminal of gcH2A-4 and gcH2A-11, we speculate the C-terminal tail of gcH2A-11 contributes to the binding of CLRs with gcH2A-11, which still remains to be further confirmed. Notably, the immunization with the engineered *S. cerevisiae* expressing gcH2A-11, which contains an additional conventional H2A sequences, can activate higher PRRs-mediated immune responses compared with gcH2A-4.

Several vaccines against *F. columnare* have been developed and considerable protection could be acquired. When immunized with the *F. columnare* G4cpN22 ghosts (FCGs), the relative percent survival (RPS) of grass carp in FCG group (70.9%) was significantly higher than formalin-killed *F. columnare* (FKC) group (41.9%). Compared with the fish immunized with FKC or PBS control groups, the grass carp immunized with FCG showed higher serum agglutination titers and bactericidal activity (47). When immunized with a recombinant *F. columnare* DnaK protein (rDnaK), the overall survival rate of the immunized channel catfish was 57% and 31% survival rate for the non-immunized group (48). In this study, RPS was 28.5% for the engineered *S. cerevisiae* expressing gcH2A-4 and 25% for the engineered *S. cerevisiae* expressing gcH2A-11. Since transcriptome data reveal that the engineered *S. cerevisiae* expressing gcH2A-4 and gcH2A-11 can activate many genes involved in antibacterial and antiviral signaling pathways, it is worthwhile to further investigate whether the engineered *S. cerevisiae* expressing gcH2A-4 and gcH2A-11 also play an immunoprotective role in viral infection.

In conclusion, we screened and obtained 7 grass carp histone H2A variants with the antibacterial activity against *F. columnare* infection. Furthermore, 2 histone H2A variants gcH2A-4 and gcH2A-11 were expressed in *S. cerevisiae* eukaryotic system, and their immunoprotective effects against *F. columnare* infection were evaluated in grass carp. The present study reveals that the recombinant *S. cerevisiae* expressing gcH2A-4 or gcH2A-11 can effectively evoke the immune response and enhance disease resistance of grass carp against *F. columnare* infection. In future work, we will further investigate the immunoprotective roles of the engineered *S. cerevisiae* expressing gcH2A-4 and gcH2A-11 in other pathogen infection.

## Data Availability Statement

The datasets presented in this study can be found in online repositories. The names of the repository/repositories and accession number(s) can be found below: https://www.ncbi.nlm.nih.gov/geo/, GSE201422.

## Ethics Statement

All animal experiments were conducted in accordance with the Guiding Principles for the Care and Use of Laboratory Animals and were approved by the Institute of Hydrobiology, Chinese Academy of Sciences.

## Author Contributions

MC conceived and designed the experiments. YY, SZ, HF, XW and JZ performed the experiments and analyzed the data. MC and JZ wrote the manuscript. MC revised the manuscript. All authors contributed to the article and approved the submitted version.

## Funding

This study was financially supported by Wuhan Application Foundation Frontier Project (2019020701011467) and the National Natural Science Foundation of China Grant (31873046).

## Conflict of Interest

The authors declare that the research was conducted in the absence of any commercial or financial relationships that could be construed as a potential conflict of interest.

## Publisher’s Note

All claims expressed in this article are solely those of the authors and do not necessarily represent those of their affiliated organizations, or those of the publisher, the editors and the reviewers. Any product that may be evaluated in this article, or claim that may be made by its manufacturer, is not guaranteed or endorsed by the publisher.
